# Supplementary material for: Sodium-Glucose Co-Transporter 2 Inhibitors Reduce Macular Edema in Patients with Diabetes mellitus
Source: Life (Basel). 2022 May 6;12(5):692. doi: 10.3390/life12050692 (PMC9146993; doi:10.3390/life12050692)
Supplement: Supplementary file 1 [file life-12-00692-s001.zip › Supplementary Table S1.pdf]

Supplementary Table S1. Clinical data of each case at baseline in SGLT2i group

Case	Age (year)	Sex M/F	Eye R/L	Agents added or changed to	Diabetes duration (years)	HbA1c (%)	Cre (mg/dl)	eGFR (ml/min/ 1.73m^2)	HT	DL	Pattern of DME	History of PC	CRT (μm)	BCVA (logMAR unit)	BCVA (Snellen equivalent)	IOP (mmHg)
1	69	M	R	SGLT2i	40	5.6	0.98	59.1	+	+	sponge-like	PRP	701	0.699	20/100	13
			L	SGLT2i							CME	PRP	431	0.398	20/50	13
2	48	F	R	SGLT2i	8	5.8	NA	NA	+	-	CME	PRP	365	0.301	20/40	18
			L	SGLT2i							CME	PRP	362	0.222	20/33	16
3	70	M	R	SGLT2i	25	9.9	0.82	71.2	+	-	sponge-like	focal	457	1	20/200	10
			L	SGLT2i							sponge-like	focal	498	1.398	20/500	12
4	65	M	L	SGLT2i	15	7.1	1.17	49.8	+	+	sponge-like	none	363	0.222	20/33	17
5	70	M	R	SGLT2i	18	7.2	0.79	76.1	+	-	CME	PRP	471	0	20/20	14
6	66	F	R	SGLT2i	19	6.2	0.91	48.0	-	-	sponge-like	PRP	521	0.824	20/133	19
7	49	M	L	SGLT2i	15	6.4	0.69	95.8	-	-	sponge-like	PRP	356	0.097	20/25	17
8	61	F	R	SGLT2i	45	6.0	0.59	70.6	-	-	CME	PRP	418	0.155	20/29	16
			L	SGLT2i							sponge-like	PRP	489	1	20/200	9
9	62	M	L	SGLT2i	10	10.4	1.15	50.5	-	-	sponge-like	PRP	431	0.523	20/67	14
10	71	F	R	SGLT2i	8	6.9	NA	96.0	+	-	sponge-like	none	378	0.046	20/22	15
			L	SGLT2i							CME	none	426	0.301	20/40	14
11	72	F	R	SGLT2i	22	6.5	NA	87.8	-	-	sponge-like	none	370	0	20/20	9
12	59	M	L	SGLT2i	9	6.9	0.95	64.7	+	-	sponge-like	PRP	334	0.097	20/25	17
13	51	F	L	SGLT2i	1	7.1	0.42	119.8	-	-	sponge-like	none	402	0	20/20	12
14	72	M	R	SGLT2i	1	7.3	0.79	73.6	-	-	sponge-like	none	331	-0.079	20/17	13
15	50	M	L	SGLT2i	2	6.6	0.83	77.4	+	+	sponge-like	none	422	0.155	20/29	15
16	60	F	L	SGLT2i	5	6.9	0.54	87.7	+	-	CME	focal	409	0.046	20/22	17
17	79	F	L	SGLT2i	5	7.5	0.57	75.7	+	+	CME	none	506	0.301	20/40	12
18	47	M	R	SGLT2i	3	8.5	0.76	83.2	+	+	sponge-like	none	373	-0.079	20/17	20
19	50	M	L	SGLT2i	20	7.1	0.62	106.5	-	-	sponge-like	PRP	346	-0.079	20/17	17
Mean ± SD	62.3 ± 8.6				14.3 ± 12.4	7.18 ± 1.23	0.79 ± 0.21	77.0 ± 19.8					423.3 ± 79.8	0.314 ± 0.389		14.3 ± 2.8

Cre = serum creatinine; eGFR = estimated glomerular filtration rate; HT = Systemic hypertension; DL = Dyslipidemia; DME = diabetic macular edema; PC = photocoagulation; CRT = central retinal thickness; BCVA = best corrected visual acuity; logMAR = logarithm of minimum angle of resolution; IOP = intra ocular pressure; SGLT2i = sodium-glucose co-transporter 2 inhibitors; PRP = pan-retinal photocoagulation; CME = cystoid macular edema; NA = Not available; SD = standard deviation.
