# Supplementary material for: Sodium-Glucose Co-Transporter 2 Inhibitors Reduce Macular Edema in Patients with Diabetes mellitus
Source: Life (Basel). 2022 May 6;12(5):692. doi: 10.3390/life12050692 (PMC9146993; doi:10.3390/life12050692)
Supplement: Supplementary file 1 [file life-12-00692-s001.zip › Supplementary Table S2.pdf]

Supplementary Table S2. Clinical data of each case at baseline in non-SGLT2i group

Case	Age (year)	Sex M/F	Eye R/L	Agents added or changed to	Diabetes duration (years)	HbA1c (%)	Cre (mg/dl)	eGFR (ml/min/ 1.73m^2)	HT	DL	Pattern of DME	History of PC	CRT (μm)	BCVA (logMAR unit)	BCVA (Snellen equivalent)	IOP (mmHg)
1	71	F	R	SU	21	6.6	0.50	90.0	-	-	CME	PRP	335	0.155	20/29	8
2	65	F	R	SU	2	6.7	0.57	79.7	+	-	sponge-like	none	313	-0.079	20/17	11
3	52	M	L	SU	2	6.7	0.79	80.8	+	-	sponge-like	focal	585	0.699	20/100	17
4	52	M	R	SU	1	6.5	0.71	90.8	+	+	sponge-like	none	323	-0.079	20/17	18
5	60	F	R	SU	15	8.9	0.63	73.4	+	+	sponge-like	none	304	0	20/20	14
6	58	F	R	GLP-1	8	6.7	0.48	99.8	-	+	SRD	none	511	0.301	20/40	16
7	78	F	R	DPP-4i	1	5.8	0.68	62.6	-	-	sponge-like	none	479	0.097	20/25	11
8	52	M	R	DPP-4i	23	10.7	1.27	47.8	-	+	sponge-like	PRP	337	0.699	20/100	8
			L	DPP-4i							CME	PRP	463	-0.079	20/17	16
9	53	M	R	metformin	24	8.7	1.03	59.8	-	+	sponge-like	PRP	359	0.398	20/50	10
			L	metformin						-	CME	PRP	439	0	20/20	12
10	62	M	L	GLP-1	7	6.5	1.48	38.6	-	-	CME	none	335	-0.079	20/17	9
11	61	M	R	DPP-4i	5	6.0	1.25	46.7	-	-	sponge-like	none	305	-0.079	20/17	9
			L	DPP-4i					-	-	CME	none	341	0.097	20/25	8
12	72	F	R	αGI + glinide	9	7.6	0.56	79.2	-	-	sponge-like	none	363	-0.079	20/17	10
13	62	M	R	TZD	3	11.4	0.65	94.2	-	-	sponge-like	none	293	0	20/20	17
			L	TZD							CME	none	362	0.155	20/29	17
14	65	F	R	GLP-1	10	8.1	0.63	71.7	-	-	sponge-like	none	356	0.222	20/33	14
			L	GLP-1							sponge-like	none	308	0.301	20/40	12
Mean ± SD	61.6 ± 7.8				9.4 ± 7.9	7.64 ± 1.67	0.80 ± 0.31	72.5 ± 18.3					374.3 ± 79.4	0.139 ± 0.241		12.5 ± 3.4

Cre = serum creatinine; eGFR = estimated glomerular filtration rate; HT = Systemic hypertension; DL = Dyslipidemia; DME = diabetic macular edema; PC = photocoagulation; CRT = central retinal thickness; BCVA = best corrected visual acuit y; logMAR = logarithm of minimum angle of resolution; IOP = intra ocular pressure; SU = Sulfonylurea; CME = cystoid macular edema; PRP = pan-retinal photocoagulation; GLP-1 = Glucagon-like peptide-1; SRD = serous retinal detachment; DPP-4i = Dipeptidyl peptidase-4 inhibitor; αGI =α-glucosidase inhibitor; TZD = thiazolidinediones; SD = standard deviation.
